# IoT Platform for Energy Sustainability in University Campuses

**DOI:** 10.3390/s21020357

**Published:** 2021-01-07

**Authors:** Pedro Moura, José Ignacio Moreno, Gregorio López López, Manuel Alvarez-Campana

**Affiliations:** 1 Institute of Systems and Robotics, University of Coimbra, 3030-290 Coimbra, Portugal; 2 Dpto. Ingeniería de Sistemas Telemáticos, ETSI Telecomunicación, Universidad Politécnica de Madrid, 28040 Madrid, Spain; joseignacio.moreno@upm.es (J.I.M.); manuel.alvarez-campana@upm.es (M.A.-C.); 3 Institute for Research in Technology, ICAI, Comillas Pontifical University, 28015 Madrid, Spain; gllopez@comillas.edu

**Keywords:** IoT platform, university campus, solar photovoltaics, monitoring and control, building refurbishing, smart buildings, nZEB

## Abstract

University campuses are normally constituted of large buildings responsible for high energy demand, and are also important as demonstration sites for new technologies and systems. This paper presents the results of achieving energy sustainability in a testbed composed of a set of four buildings that constitute the Telecommunications Engineering School of the Universidad Politécnica de Madrid. In the paper, after characterizing the consumption of university buildings for a complete year, different options to achieve more sustainable use of energy are presented, considering the integration of renewable generation sources, namely photovoltaic generation, and monitoring and controlling electricity demand. To ensure the implementation of the desired monitoring and control, an internet of things (IoT) platform based on wireless sensor network (WSN) infrastructure was designed and installed. Such a platform supports a smart system to control the heating, ventilation, and air conditioning (HVAC) and lighting systems in buildings. Furthermore, the paper presents the developed IoT-based platform, as well as the implemented services. As a result, the paper illustrates how providing old existing buildings with the appropriate technology can contribute to the objective of transforming such buildings into nearly zero-energy buildings (nZEB) at a low cost.

## 1. Introduction

University campuses are normally constituted of large buildings responsible for high energy demand. The challenges associated with high energy costs and environmental impacts are clear motivations to achieve efficiency and sustainability goals. Additionally, University buildings are important as demonstration sites for new technologies and systems. This paper presents the considered options of energy sustainability in the set of four buildings that constitute the Telecommunications Engineering School of the Universidad Politécnica de Madrid (ETSIT-UPM). Located at the Campus of International Excellence in Moncloa (University City of Madrid), ETSIT is a national and international reference center in the field of teaching, research and technological development and innovation in the area of information and communications technology (ICT).

The development of ETSIT’s teaching and research activity entails a significant consumption of energy resources. Therefore, in order to achieve sustainability goals, the main options are the reduction in energy consumption and the integration of renewable generation. These are the two main pillars that can enable a high reduction in net electricity consumption in order to transform the buildings into nearly zero-energy buildings (nZEB). However, in public buildings, one major challenge is to ensure the energy services in different areas of the building, taking into account the variable schedule of activities, presence of users and weather conditions, while avoiding wasting energy when such energy services are not needed. Another major challenge imposed by the large-scale integration of renewables is the need to increase the matching between local generation and demand, in order to increase the self-consumption of the local generation to boost the technical and economic advantages with such generation.

Monitoring and control platforms therefore have a crucial role in addressing such challenges, ensuring not only the monitoring of the global electricity demand and generation, but also the individual consumption of different building areas and electrical systems, as well as other relevant information such as weather data and the presence of users to be used in load control. There are options of monitoring and control platforms available on the market, but in most cases, its integration in old existing buildings is not easy and such platforms have a high initial cost that reduces the economic advantages potentially achieved with the monitoring and control. Another relevant barrier of such systems is the limited capability to include novel energy services—able to ensure the control of electrical systems not only to achieve energy savings, but also to provide flexibility able to contribute to increasing the self-consumption of the local generation.

Therefore, this paper deals with the options to achieve more sustainable uses of energy, considering the integration of renewable generation sources and the use of a smart system to control loads. In this case, the use of photovoltaic (PV) technology was considered because it was the most cost-effective option for this location and building type, however other generation options can be used, being the control based on the total generation instead of the PV generation. To ensure such monitoring and control, a low-cost IoT-based platform based on wireless sensor networks (WSN) has been deployed to manage and control electrical systems, with a special focus on heating, ventilation, and air conditioning (HVAC) and lighting. Such smart control ensures not only a reduction in energy use and associated costs, but also an effective integration of the PV generation (or other local energy sources) using the management of loads to increase the matching between the local generation and demand, therefore being crucial to transform old buildings in nearly zero-energy buildings. Furthermore, the paper presents the developed IoT-based platform, as well as the implemented services.

The remainder of the paper is structured as follows. Section 2 assesses the related works and Section 3 presents the characterization of the building and its electricity demand. In Section 4, the sustainable energy options to be implemented are assessed, and a PV generation system is designed to ensure the integration of renewable generation and assess the options to ensure the monitoring and control of the existing demand. Then, Section 5 presents the IoT-based platform deployed on the campus, and Section 6 describes the implemented energy services, as well as future options to increase the level of services using the actual platform. Finally, Section 7 summarizes the paper, emphasizing its main conclusions.

## 2. Related Works

Smart campuses have been attracting increasing attention, mainly because they represent an ideal environment to develop, evaluate and validate smart city and smart building solutions before applying them at larger scales. As a token of this, ref [1] reports that the published articles on smart campuses grew from 19,000 in 2010 to 28,400 in 2017; there is a vast body of work proposing the implementation of smart and sustainability options in University campuses, from the energy and ICT point-of-view. Some works are focused on the implementation of energy efficiency options. For instance, ref [2] presents the envelope refurbishment and improvement of building services efficiency at the University of Brescia (Italy) and in ref [3] energy efficiency interventions and rehabilitation actions are evaluated for the University of Palermo (Italy).

The most common option is the integration of renewable energy generation. At Leuphana University of Lueneburg (Germany), the heat and electricity supply was changed to renewable energy with biomethane-powered combined-heat-and-power and PV [4]. Ref [5] presents the main steps in the design of large-scale PV at the Technical University of Crete (Greece). The microgrid with several renewable energy options, such as PV, solar water heating, wind power and hydrogen, implemented in the Al Akhawayn University (Morocco) is presented in ref [6].

Other works have proposed the use of energy efficiency options together with the integration of renewables. In ref [7], the cost-effectiveness of converting the University of Dayton (USA) to a fully-electrified, renewably powered, carbon-neutral campus is evaluated, and in ref [8] a microgrid is assessed for the University of California, San Diego (USA), integrating renewable generation with peak load shifting.

Other works have designed microgrids with energy storage for the integration of renewable generation. In ref [9], a microgrid with energy storage is proposed for the Savona Campus of the Genoa University (Italy), and in ref [10] a PV and a battery storage system is proposed for the American University of Beirut (Lebanon). In ref [11], the design of a PV and energy storage system, integrated with energy efficiency technologies is conducted to achieve a near zero-energy building at the University of Coimbra (Portugal). However, such works do not implement ICT platforms to ensure the real-time monitoring and control of the loads.

Other papers have proposed ICT platforms, but without ensuring any implementation. For instance, in ref [12], the interconnection of sensor networks and energy management systems integrated with the campus IT infrastructure was designed for the Covenant University (Nigeria). In ref [13], an IoT-based smart campus model was presented for the University of Rajshahi (Bangladesh), but only the smart environment monitoring and EV charging was implemented. Other works have developed ICT platforms, but not focused on energy management. Ref [14] presents a platform for authentication and data analysis, in order to assess behavior and daily habits in Shandong Normal University (China). Ref [15] presents the IoT platform deployed across the engineering schools of the Universidad Politécnica de Madrid (Spain) monitoring people flow and environmental parameters. Other works describe the implementation of a platform, but focused on the distribution of energy between buildings, such as in the Trieste University Campus (Italy) [16].

The monitoring and control of the energy demand and generation in buildings are addressed in several works. Ref [17] presents the adaptation of the physical energy supply systems of the Chalmers University of Technology (Sweden) to integrate the communication and control set-ups that provide the technical requirements for smart grid interoperability. Ref [18] presents the wireless sensor nodes and the monitorization of load for the Jadavpur University Saltlake Campus (India). In ref [19], an efficient web-based energy management system is presented for the University of Crete (Greece), which manages the campus buildings and spaces of public use in an energy-efficient way, monitors the energy load, and performs energy analysis for each building and for the campus as a whole. Ref [20] provides a review of the role of technologies such as IoT, blockchain, or edge and fog computing as an enabler for smart campuses.

In ref [21], a general framework of the different layers of a smart-campus is presented, including the main technological infrastructures associated with their implementation applied to the University of Malaga in Spain. In ref [1], analysis and design of service discovery and perceptual data fusion algorithms are applied to situational awareness in the smart campus, ref [22] shows how multidimensional situational information fusion methods can be used to perform intelligent control for energy saving on campus, while ref [23] focuses on on-cloud and big data architecture to support university campuses. Ref [24] provides an in-depth review of the different types of internet of energy-based building energy management systems, such as energy routers, storage systems and materials, as well as renewable sources, and ref [25] illustrates how IoT platforms can be used at the school level to promote energy-saving behaviors supported by the data gathered by such platforms. Finally, ref [26] presents an architecture for the data management of smart buildings, including data from a validation testbed.

Table 1 presents a comparison of the main implementations identified in the literature for university campuses (excluding reviews and other works without implementation in universities).

As can be concluded, the related works are usually focused only on energy or ICT issues without presenting an integrated approach for both aspects. Additionally, the implemented ICT platforms are usually for the monitoring of energy demand at the building or campus level, without individual monitoring of loads and their control. The main problem to be solved is the design and implementation of a low-cost IoT platform, ensuring the interoperability between existent low-cost devices while ensuring the implementation of novel energy services able to ensure energy savings, and the matching between local generation and demand. The main contributions of this paper are an integrative perspective that includes the energy option to achieve a sustainable energy campus, but also the deployment of an IoT platform that ensures the necessary condition to implement smart energy services. Such a platform not only provides global information about energy demand and generation at the building and campus level, but also ensures the monitoring and control of the main loads, namely lighting and HVAC. With such services, it is not only possible to decrease the energy consumption and associated costs, but also to control the demand to increase the matching between local generation and demand and, in the future, to implement demand response actions, providing services to the grid.

## 3. Building Characterization

The four buildings of the ETSIT are constituted of a series of classrooms, teaching or research laboratories, and offices of professors and researchers:

- Building A (Sanz Mancebo)—building with the largest number of classrooms and student laboratories, as well as some teachers’ offices, main hall, meeting room, evaluation rooms, assembly hall, library, canteen, student clubs, secretariat, and directors’ board.
- Building B (García Redondo)—building with other classrooms, some student laboratories and numerous research laboratories and professors’ offices (the building is connected to building A using an elevated corridor known as “the accelerator”).
- Building C (López Araujo)—this building communicates with building B and is mainly constituted of research laboratories, and professors’ offices, but also by the museum and a second assembly hall (attached to this building is the Solar Energy Institute).
- Building D—this is the most recent building, it is connected to building A and was conceived in part as a business incubator, but it also contains more research laboratories and offices.

**Figure 1:**
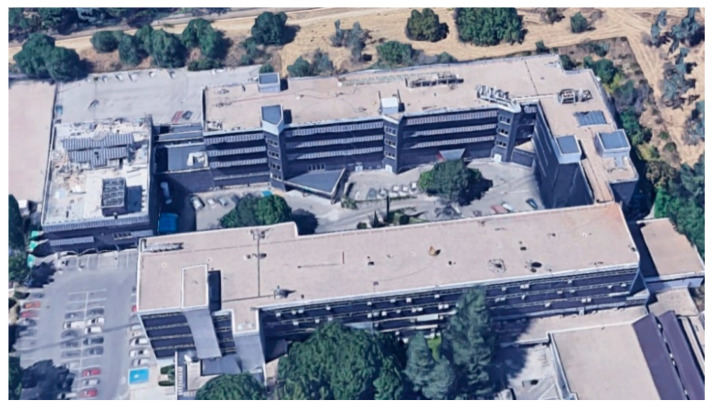
Buildings B and C.

**Figure 2:**
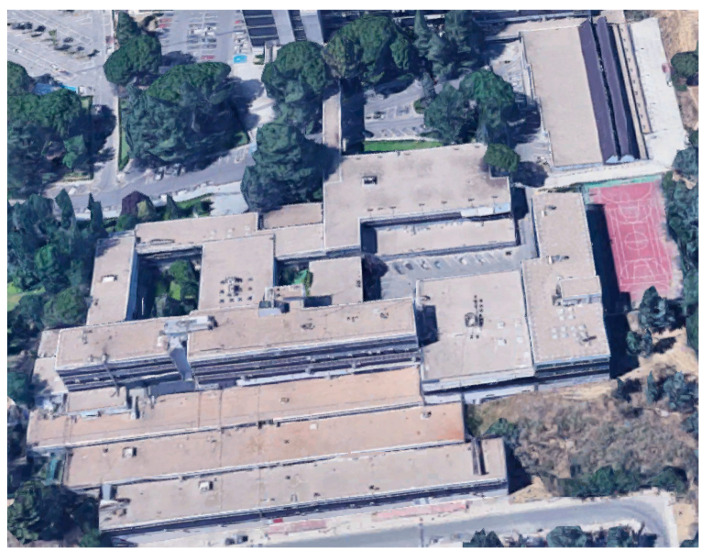
Buildings A and D.

Figure 1 presents buildings B and C, and Figure 2 presents buildings A and D.

**Figure 3:**
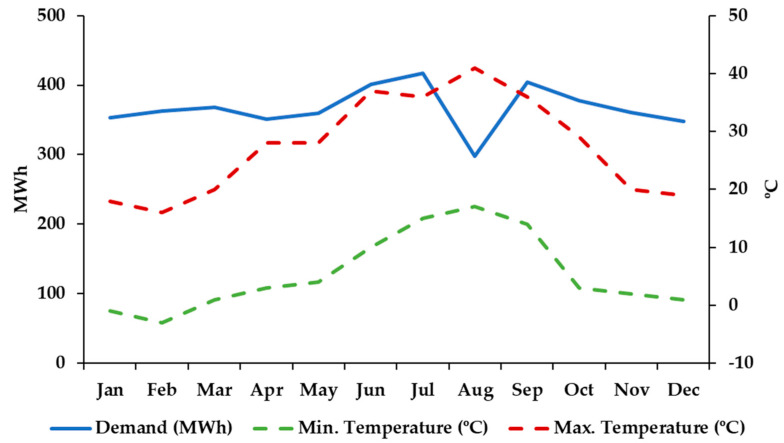
Monthly electricity demand, minimum (Tm) and maximum temperature (TM) in 2018.

In 2019, the buildings presented an electricity demand of about 4.4 TWh/year. The buildings have an area of 44.020 m^2^, therefore this corresponds to a specific consumption of 99.96 kWh/m^2^ year. Figure 3 presents the variation of the electricity consumption of the building during the year. As can be seen, the consumption does not present major variations, except for August, which presents a much lower consumption due to the holiday period. The maximum electricity consumption (in July) is 40% higher than the minimum (in August). However, excluding August, such variation is reduced to only 20% (the standard deviation is reduced from 31.46 MWh to 23.75 MWh). The highest consumptions are achieved in the summer, due to the high impact of the cooling demands. This is confirmed by observing the variations of the average minimum and maximum temperature, with an increase in the maximum temperature associated with a demand increase. The same occurs, but with a lower impact, on the low values of minimum temperature.

**Figure 4:**
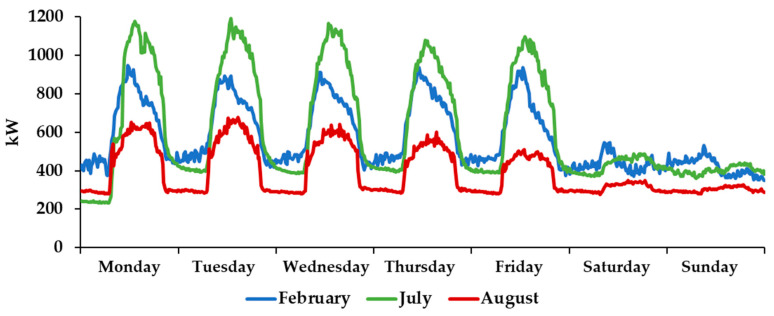
Load profiles during one week in February, July and August.

Figure 4 presents examples of the load profiles during the first week of February, July, and August. As can be seen, the electricity demand during the weekend is much lower than on weekdays, and the demand during August is much lower than in other months due to the summer holidays. The high demand at night is justified by the use of the building by students and researchers during such a period.

## 4. Sustainable Energy Options

Several options were assessed to achieve energy sustainability and zero-energy levels in the building, namely the integration of renewable energy generation, using solar photovoltaic (PV) generation and energy storage with lithium-ion batteries, as well as the monitoring and control of the main loads.

### 4.1. Renewable Energy Integration

To evaluate the potential for PV generation of the ETSIT buildings, the available areas and orientations were measured using Google Earth. Considering the use of 50% of the available roof area, to ensure the spacing to avoid shading and enable other uses of the roof, it is deemed possible to install 3856 panels. There are already PV panels installed on three different levels of the façade of building C, and the generation of such panels was taken into account in the assessment and a similar structure was therefore considered for buildings B and A. Table 2 presents the total number of panels.

The PV system was then designed and simulated using Sunny Design [27], considering the meteorological data of Madrid, using an annual total global irradiation of 1655.16 kWh/m^2^a. The buildings are not perfectly oriented to the South, but there is only an angle of 7.8°. A tilt angle of 38° was considered, with the PV panels with the same orientation of the building, facilitating their integration [28], because a 7.8° angle only reduces the average generation by less than 0.5%, as was evaluated using Sunny Design [27]. The main characteristics of the designed PV system are presented in Table 3. As can be seen, the system has a peak power of about 1.22 MWp and an average annual energy generation of 1935 MWh.

### 4.2. Matching between the Renewable Generation and Demand

To evaluate the self-consumption of the generated energy, the electricity consumption records of the building in 2019 were used with a time interval of 15 min. The simulations of PV generation are typically performed with one year of data, because the variation of average solar radiation between years is very low and one year of data are enough to evaluate the seasonal impact during the year. If the demand presents a high variation between years, there is a relevant impact on the evaluation of self-consumption. However, this is not the case of the studied building, because in a university the used equipment and hours of operation do not present major variations. Therefore, the electricity demand from the previous years does not present any significant variation, meaning that one year of data is enough information for the evaluation.

Table 4 presents the distribution of the PV generation and assesses the self-consumption, self-generation, and self-sufficiency in the buildings. As can be seen, 83% of the generated energy is used in the building as self-consumption, and 17% has to be injected into the grid (due to the high generation in periods of low demand). The generation is equivalent to 44% of the demand, but to compensate for the grid feed-in, the energy purchased from the grid is 63.5%, leading to a self-sufficiency of 36.5%.

**Figure 5:**
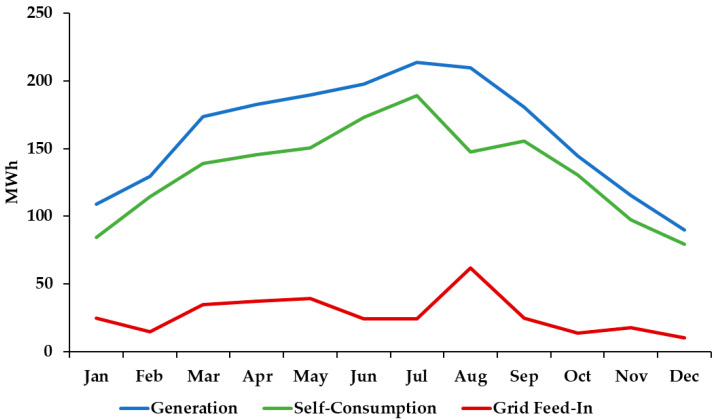
Variation of the PV generation, self-consumption and grid feed-in.

Figure 5 presents the variation of the PV generation, self-consumption, and grid feed-in throughout the year. As expected, the generation is higher during summer, leading to higher self-consumption levels. Regarding the grid feed-in, there is a high increase in August due to the low demand and high generation level.

With the objective to increase the matching between PV generation and demand, therefore reducing the grid feed-in, an energy storage system with lithium-ion batteries with a total capacity of 480 kWh with a minimum state of charge of 20% and a round trip efficiency of 85% was considered, with the impact presented in Table 5. As can be seen, despite the high energy storage capacity, the impact on self-consumption is not high, only achieving an increase of 3.6%. This is justified by the low daily use of the battery.

Unlike what happens in residential buildings, where there is typically a daily cycle, charging the surplus during the day to be used at night, in this case, most days do not present a generation surplus. The generation surplus is concentrated on the weekend, with the battery mainly used to store such a surplus in order to be used on Monday. This leads to an annual nominal energy throughput of the battery of only 139. Therefore, only from the self-consumption point-of-view, such a storage system is not cost-effective. However, the capacity not used to store generation surplus can be used to store electricity from the grid in periods of lower cost, to minimize the costs with the purchased electricity, as presented in ref [11].

A large lithium-ion battery system is not cost-effective to ensure the matching between PV generation and the electricity demand; other options should be considered. Therefore, an effective option with a lower cost is to implement the control of part of the demand, being able to adjust the demand based on the PV generation availability in order to concentrate the demand in periods with high availability of generation. The ideal load to implement such a service is the HVAC system, because it is possible to turn off some HVAC devices, during short periods, without a major impact on the thermal comfort in the building (e.g., periods of reduced generation due to the passage of clouds). The control of such loads can also ensure the minimization of costs with electricity imported from the grid, as in the case of energy storage, e.g., by ensuring the pre-heating of the building to take advantage of the lower tariffs in the early morning or concentrating the heating in periods of high PV generation levels.

Regarding the future potential impacts of climate change, several studies have assessed the impact of climate changes on renewable generation. In ref [29], the most relevant quantity projections of PV generation were reviewed. The conclusions are often focused on specific areas, because climate changes can lead to increased generation in some regions and decreases in others. However, the reviewed studies predict an increasing PV output in Europe, and mainly in southern Europe, with an increase of up to 10%. Therefore, in the location of the assessed building, climate changes are expected to lead to a higher global generation, and the daily and monthly variability will not substantially be affected [30].

The uncertainties caused by climate changes are not much higher than the actual uncertainties due to the variation of weather conditions between seasons or days. As presented in Figure 5, the generation level has a seasonal variation leading to a different level of self-consumption or even to the injection into the grid of generation surplus. This is a grid-connected building and not an isolated system, therefore any major deviation in the total generation is compensated by the electrical grid. Rapid variations of the generation due to clouds can be compensated with control of demand, but variations during a higher time frame (e.g., a full day with low or high generation) need to be compensated by the grid, increasing the electricity imported from the grid in the case of lower generation or increasing the grid feed-in with high generation levels.

From the energy efficiency point-of-view, the control and monitoring of loads are also crucial. As can be seen in Figure 4, the building presents a high baseload which indicates that a large number of loads are permanently in operation, even when not needed. Therefore, it is important to individually monitor the main loads, to remotely identify when they are used or not to contribute to more effective management. Additionally, the remote control of such loads can ensure that they are turned off in the periods when they are not supposed to operate. For this objective, the most important loads are lighting and HVAC, which typically ensure more than 65% of the electricity consumption in this type of buildings [11]. It is crucial to install technology to enable the individual monitoring of such loads, as well as to its remote control to achieve energy savings and enable its control to ensure a higher matching between local generation and demand, take advantage of periods with lower tariffs, and in the future even provide demand response services to the grid. Therefore, a new IoT platform to ensure such monitoring and control was developed, as presented in Section 5.

## 5. IoT Platform

The internet of things (IoT) allows the deployment of platforms and infrastructure to monitor and actuate different elements. In this sense, energy sustainability is one of the key application sectors. This section describes an energy monitoring and control platform applied to a university building, over more than 30 years. The system to be deployed will have to support the following requirements:

- Low-cost of implementation. Due to the application in an old building, the solution should be cost-effective and with minimal impact during installation and deployment on the current systems.
- Individual monitoring and control support of different electrical systems, including lighting and HVAC systems. The system should monitor and control individual devices (e.g., lights of specific areas), as well as a complete subsystem (e.g., all lights on a floor, all HVAC, etc.) to ensure dynamic granularity.
- Automation of the control of the electrical systems, requiring low human interaction. The system should be able to support the definition of pre-configurated behavior, as well as manual interventions.
- Evaluation of energy consumption. The system should report the power and evaluate the energy consumption from the different monitored systems.
- Integration with the current infrastructure in terms of communications and energy. The IoT platform to be deployed should not involve any specific network technology or new investment on energy deployments, but will be integrated with the existent infrastructure such as Wi-Fi and differential switches.

This section describes the architecture design, as well as platform deployment, setup and evaluation over a real infrastructure located at ETSI Telecomunicación (UPM-Madrid) to manage lighting and HVAC systems. The objective is to evaluate the platform and the application of policy rules to centralize control and energy consumption over the testbed. This infrastructure has been developed to complete the Smart Moncloa Campus infrastructure.

### 5.1. Smart CEI Moncloa

The Smart CEI (Campus of International Excellence) Moncloa was deployed within the context of the UPM City of the Future initiative. It is an IoT platform that currently offers two services [15]:

- people flow monitoring;
- and environmental monitoring.

There are several techniques to perform people flow monitoring (e.g., based on radiofrequency, cellular technologies, GPS, Bluetooth, or Wi-Fi) [31]. In the case of Smart CEI Moncloa, this service is provided employing Wi-Fi tracking, using low-cost Wi-Fi sensors based on Raspberry Pi. The software running in these sensors has been specifically developed for the Smart CEI Moncloa platform, and allows the scanning of all Wi-Fi channels and recording the MAC addresses of the Wi-Fi-compliant devices (e.g., smartphones, tablets, laptops) in the region of coverage, storing them appropriately anonymized. To be more precise, these Wi-Fi sensors scan each of the Wi-Fi channels from both the 2.4 GHz and the 5 GHz bands during a configurable amount of time (currently, 250 ms), read the header of the radio IEEE 802.11 packets (e.g., data packets or probe requests) in its region of coverage, record the sender MAC addresses, and store a hash of them.

For the environmental monitoring service, the Smart Citizen Kit (SCK) [32] is used. The SCK is based on Arduino, and incorporates a shield board that includes sensors for temperature, humidity, light, noise level, and air quality (notably, CO and NO_2_). These devices are adapted to be able to take these measurements both indoors and outdoors.

**Figure 6:**
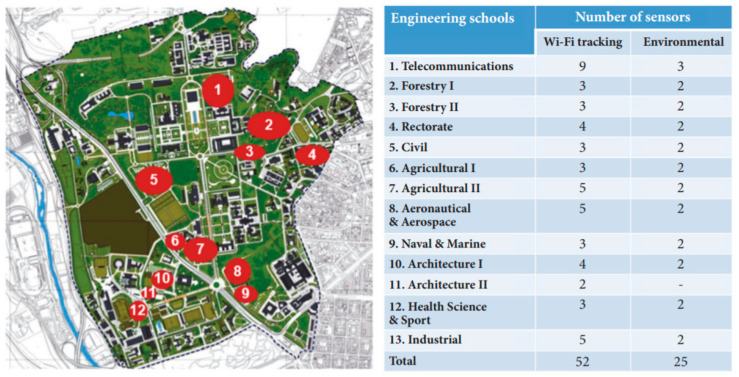
Summary of the sensors currently deployed in Smart CEI Moncloa [15].

As is shown in Figure 6, the Smart CEI Moncloa currently includes 52 Wi-Fi sensors (nine of them deployed in the ETSIT-UPM) and 25 environmental sensors (three of them deployed in the ETSIT-UPM). The Smart CEI Moncloa has been used and further developed in a remarkable number of BSc and MSc theses, as well as in research works. Among the latter, it is worth mentioning that, where the huge amount of data are stored from the Wi-Fi tracking devices of the ETSIT-UPM during a whole year, this information is used for analyzing time and occupancy, the position of people, and the identification of common behaviors [31].

Based on occupancy data stored, the system will be able to actuate over the lights to save energy during periods without classes where nobody is tracked. At the same time, environmental sensors will provide the right information to actuate over the window blinds and motors awning to save energy on sunny days or cloudy days by opening or closing them and reducing the lighting use. Similar actions can be performed by using air quality sensors to force the exchange of clean air from outside by switching on the air pumps of the main rooms.

### 5.2. Demand Monitoring and Control

**Figure 7:**
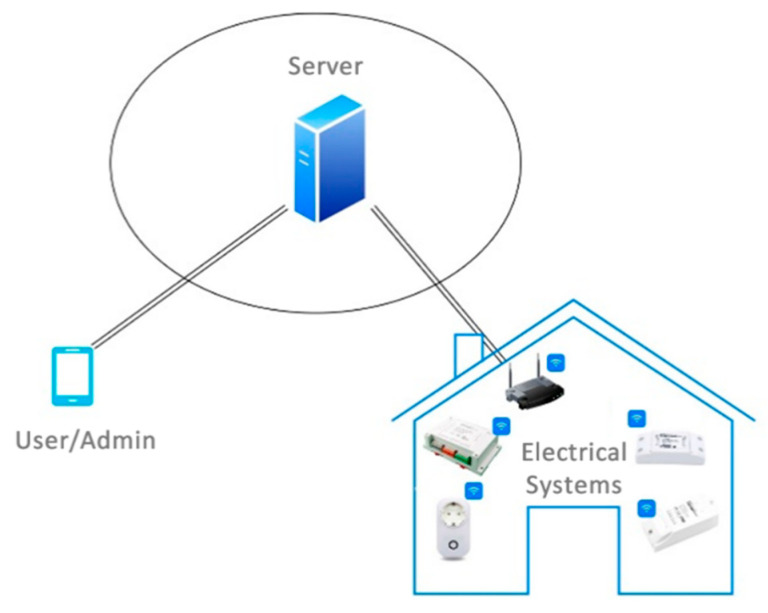
IoT system architecture.

The main architecture is depicted in Figure 7, and is composed of three main subsystems:

- Sensors, which support the acquisition of electrical consumption parameters, as well as its control by switching the status (on/off). These sensors have a low impact on the current infrastructure because they are installed in the current electrical cabinets and they are managed by using the Wi-Fi network with current coverage over the complete building.
- The server, which manages the whole system by storing parameters received from electrical systems and its control policies. The server is the brain of the IoT system because it maintains the status of the different sensors, and, based on the policy rules, will apply automatic actions. The server will manage the communication protocols and procedures to retrieve information and apply actions. This procedure will be based on a publish/subscribe interaction (message queuing telemetry transport protocol), as described later.
- User interaction, which includes two external different groups of users according to the capability to update or apply control policies. In this case, a normal user will be able to manually switch elements, whereas the administrator will be able to create policies for automatization depending on the annual calendar, holidays, summer/wintertime, environmental sensors, user tracking, etc.

For the electrical systems, firstly, the different wireless communications technologies to support the transmission of information to such systems were evaluated, as shown in Table 6.

**Figure 8:**
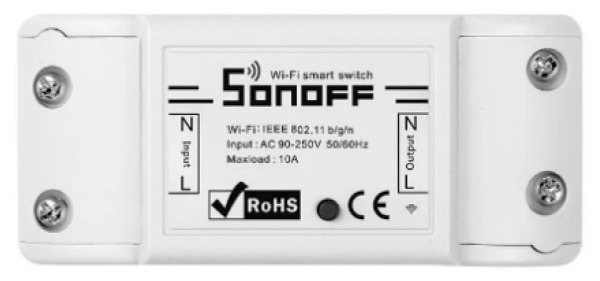
Sonoff Wi-fi Smart Switch.

**Figure 9:**
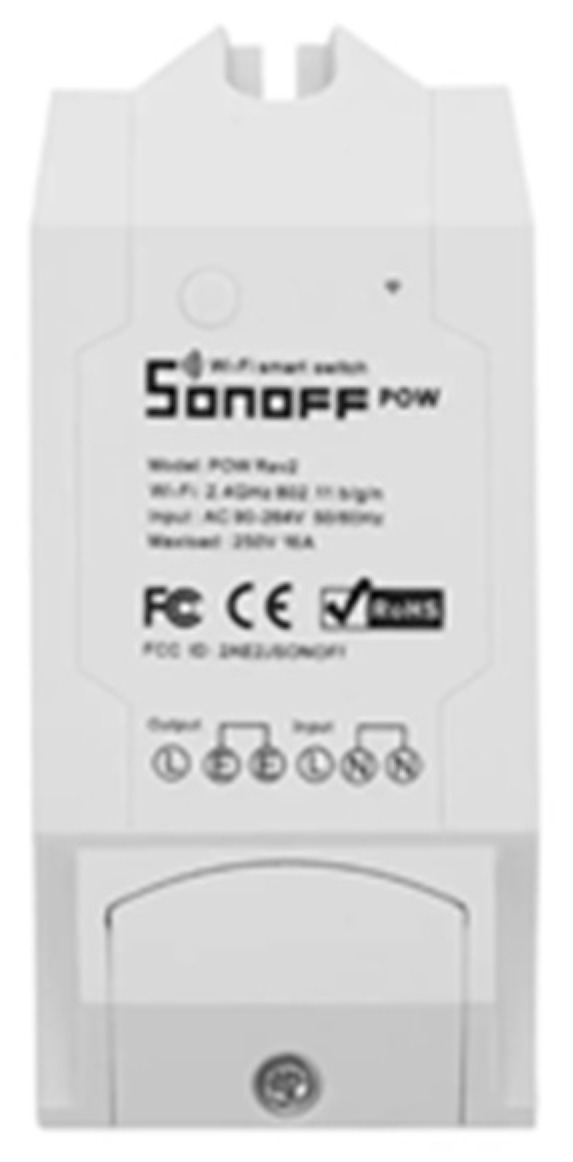
Sonoff Pow R2.

**Figure 10:**
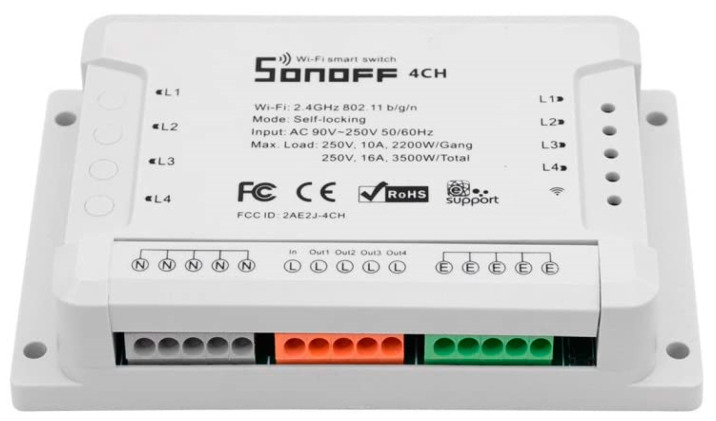
Sonoff 4CH.

In this case, the selected option was Wi-Fi because all University buildings have 100% Wi-Fi coverage. This coverage supports Eduroam [34] and besides, UPM has deployed a specific SSID for IoT deployment called “Servicios UPM”. Based on this decision, low-cost sensors from Sonoff were selected. These elements are based on chip ESP8266 which can change the firmware to support its management using local servers. There is a broad range of products from Sonoff [35], with the most relevant products used in the platform being:

- Sonoff Wi-Fi Smart Switch: This is the basic sensor (Figure 8), with the capability just to support an electrical switch and a maximum current of 10 A. The Wi-Fi connectivity supports 802.11 b/g/n with WPA-PSK/WPA2-PSK. Once connected to the electrical network, it sends the status of the switch to the server.
- Sonoff Pow/Pow R2: With a 15 A maximum current support, this sensor (Figure 9) in addition to the basic capabilities, allows the monitoring of energy consumption in real-time (power, voltage, and current) and supports historical information consumption (100-day daily/monthly).
- Sonoff 4CH: This sensor (Figure 10) is useful for deployment inside equipment racks because it supports DIN (Deutsches Institut für Normung) rail mounts as well as four independent control channels from one electrical input, and supports a maximum current per line of 10 A (2200 Watts) and a maximum of 16 A (3.5 kW) for the four lines.

**Figure 11:**
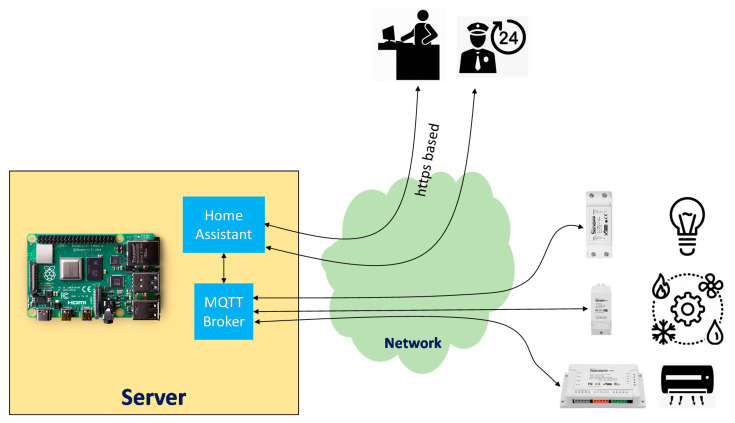
Architecture of the monitoring and control platform with MQTT (Message Queuing Telemetry Transport) protocol.

As previously mentioned, it was decided to modify the firmware of the sensors to manage them in a local server, avoiding exchanging confidential information and dependence with servers supported by the manufacturer. There are different firmware options available, such as ESPEasy [36], Tasmota [37], and ESPurna [38]. The selected option was Tasmota, because it has all firmware elements integrated and it is possible to flash new firmware following the procedure presented in ref [39]. For the server, a protocol was used to manage status messages received from all sensors and send them to a web platform to orchestrate them, as well as to apply group policies or individual actions over specific sensors in autonomous mode or manual mode. The block diagram of the server is presented in Figure 11.

**Figure 12:**
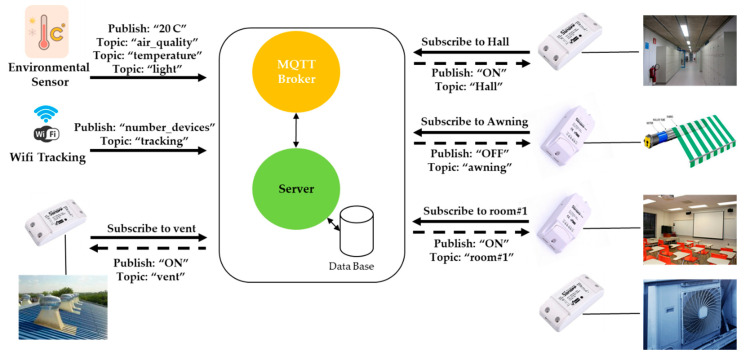
Example of MQTT Interactions.

To orchestrate message communications between server and sensors, the MQTT (message queuing telemetry transport) protocol was used. MQTT is an open and lightweight OASIS and ISO standard (ISO/IEC 20922) [40], a publish-subscribe application protocol that transports messages between devices. MQTT is the standard messaging and data communication exchange protocol for IoT [41]. The protocol is based on the publish/subscribe paradigm in which publishers and subscribers do not have any direct contact, and are unaware of their existence. The connection between publishers and subscribers is handled by a third component (MQTT broker). Clients (publishers and subscribers) connect to a MQTT broker and subscribe to a topic, and each broker can manage different topics. When a client publishes information about a topic, the broker sends this information to the appropriate set of clients that subscribe to the topic (Figure 12).

In the developed architecture, MQTT clients are composed of a set of electrical sensors, as well as the home assistant, which constitutes the central platform for control and monitoring. There are a set of current solutions to implement the MQTT broker [42], the selected being Mosquitto [43], because is suitable for the use of heterogeneous devices from low power single board computers to full servers.

**Figure 13:**
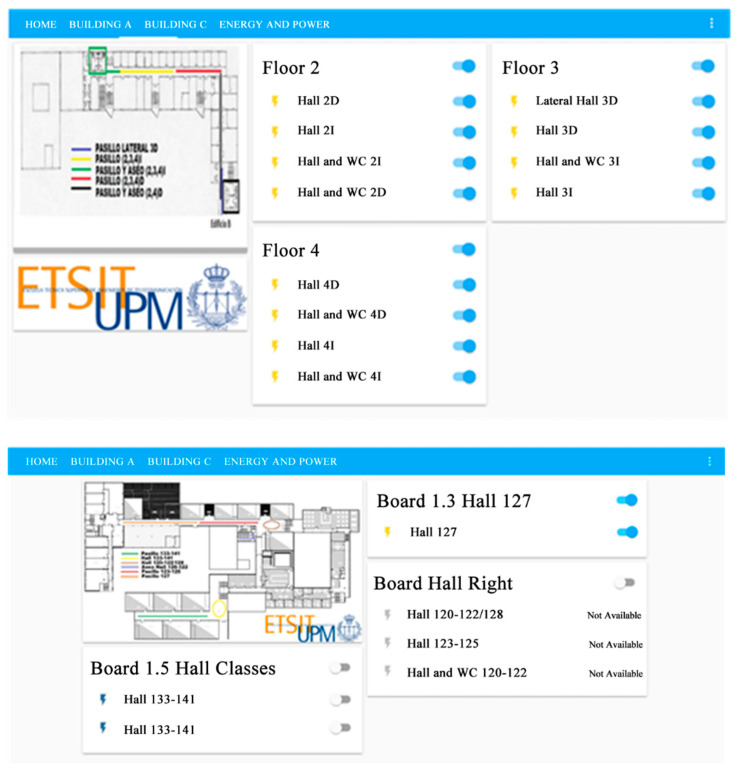
Web interface of home assistant.

As the central brain of the control and monitoring platform, home assistant [44] was used, with it being connected to the broker for receiving information from sensors by using the MQTT protocol, as well as real users throughout a web-based interface to manage the electrical monitoring platform. The home assistant was installed in Raspberry 4 hardware [45]. As a result of all this integration, the system was developed, as shown in Figure 13, in which the lighting of building B and partially from building A were monitored and controlled. Despite using current technologies, the proposed solution ensures a global architecture that ensures the interoperability between different options of sensors and controllers, and integrates data from different sources in a reliable and cost-effective way. Additionally, the developed platform enables the implementation of novel energy services that are not possible with the current technologies available on the market.

## 6. Energy Services

Several energy services are already implemented with the actual monitoring and control platform. Additionally, taking advantage of the information and services already ensured by the Smart CEI Moncloa, new energy services can be implemented using the deployed infrastructure.

### 6.1. Monitoring and Control Platform

The implemented monitoring and control platform provides the following services:

- Status monitoring. The user interface provides local or remote access, via a web-secure platform, to monitor the on/off status of each electrical system (lighting or HVAC).
- Demand monitoring. By using the information provided by POW/POW R2 sensors, the users can access the information related to the electricity consumption for each electrical system in real time (power, voltage, and current), as well as its historical information.
- Manual control. The platform allows the remote manual control of the on/off status of the electrical systems.
- Automatic control. The target is public buildings, therefore the platform allows the application of policies to manage the on/off status of lighting or HVAC systems according to a predefined schedule or other parameters, such as temperature, weather forecast, etc. This service allows the definition of an automatic behavior of the electrical systems based on a central policy.
- User/Administration profiles. The platform provides two different profiles: for a normal user (e.g., security guard, caretaker) only with access to the monitoring and basic manual control operations; or admin users with access to the automation policies, consumption registry, and the capability to modify the platform behavior.
- Multiaccess. As a web-based platform, it could be accessed by heterogeneous terminals (pc-based, smartphone) from the internal build network or the internet, allowing the monitoring and control of the building behavior from anywhere.

The innovative content is the implementation in an existent building of options that can easily ensure the individual monitoring of loads (which previously had no monitoring options) and consider them to ensure the remote control of loads, not only in terms of adapting the period of operation to the real needs of the building, but also to enable the use of such control to ensure the matching between the renewable generation and demand in the building, as well as other alternatives to reduce energy costs. This was implemented at a very low cost and can be easily replicated in other existent buildings. This represents such a relevant research area currently in Europe (as several past H2020 calls on this topic illustrate) and overseas, and universities represent such a good option to evaluate and validate proposals and ideas before applying them at larger scales.

**Figure 14:**
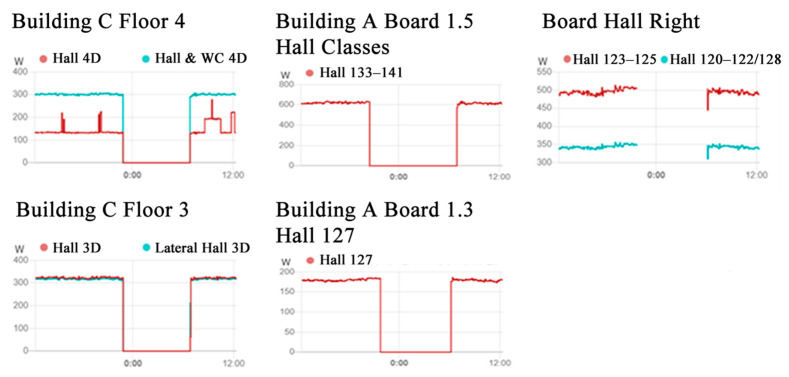
Demand profiles presented by the home assistant.

In order to manage the consumption information, the home assistant allows the creation of different profiles of power consumption over time for each of the sensors. Figure 14 presents some examples of the obtained data with the monitoring of the electricity demand in the different boards and circuits.

**Figure 15:**
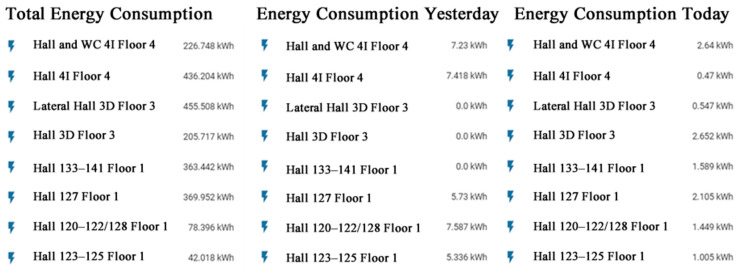
Electricity consumption for all electrical systems.

Additionally, the home assistant can also present the actual power, the accumulated energy consumption during the current day, and the accumulated energy consumption during the day before, as presented in the example of Figure 15. Such data enable not only the assessment of energy consumption to identify situations where there is unnecessary consumption to take into account in the control of the circuits, but also enable the assessment of the variation of demand to identify opportunities of load control to ensure the matching between local generation and demand and the reduction in costs.

For the pilot installation at the ETSI Telecommunications building, two phases were developed, in order to set the implemented services and validate the monitoring and control platform. In the first phase, in January 2020, 19 electrical sensors/controllers were installed to control the common lighting areas of two of the buildings. From these sensors/controllers, 11 provided a basic switching function (ensured by the Sonoff Wi-Fi Smart Switch), while eight provided not only the switching, but also the monitoring of electrical parameters (ensured by the Sonoff Pow/Pow R2). Additionally, three air conditions were monitored and controlled by Sonoff Pow P2 sensors.

During the initial phase, no control was implemented, with only the system used to retrieve information about energy consumption. In general, according to the operation time of the building, there was no consumption between 22:00 and 6:45. This behavior was produced by manually switching off the lighting systems by the building maintenance staff; the previous management only relied on manual control. Therefore, some lighting circuits were always on during working days and were not switched based on the concrete required period, because any actuation needed the displacement of staff from the helpdesk to the concrete area. For example, some lighting in teaching areas was on during the entire operation period, even if the period of classes was much shorter, or during weekends some lighting must be on 24/7 due to security reasons. Therefore, the operation period was not only adapted to the real needs, but also any failure of the staff would lead to leaving the systems running overnight or at the weekend. It was assessed that, for instance, if the lighting remained turned on during the weekend, that represented an increase of about 30% on the energy consumption during such week. Therefore, the first phase was mainly used to validate the data obtained in the monitoring platform, as well as to identify the needed control to schedule the operation of the loads according to the needs of the building.

In a second phase, an automatic configuration was implemented by using the home assistant, to adapt the operation of the lighting to the building administration policy. With this option, the building staff can control the electrical systems anytime (switching on and off) by using a simple home assistant interface from the building helpdesk, as presented in Figure 13. There is a central control accessible from the internet and intranet, which allows operating and knowing the status of each electrical system from anywhere and anytime.

In this phase, the consumption during a complete week was assessed, with such values used to tune the automatic schedule of the electrical systems, mainly by turning off some parts of the installation in low usage hours (e.g., after 20:00). The effectiveness of the control was evaluated using the monitoring data, and such control can be regularly updated and there is the capability to centralize and modify the energy policy according to summer/winter/holidays/special days. It was also possible to conclude that the selected tools were effective to monitor and control the consumption of the installation at a low price and with low impact in the current installation, because this only requires the installation of the sensor in the electrical panel after the protection switch.

### 6.2. Energy Management Services

The information gathered, not only by the Smart CEI Moncloa regarding people flow and environmental monitoring, but also the information of the total electricity demand and PV generation at the building level which is also collected by obtaining data from the smart meters [46], ensures that the required data guarantee effective implementation of the automatic control.

Using the people flow monitoring, it is possible to detect if some areas of the building do not have any users present. This information can then be used to automatically turn off the lighting and HVAC in such areas. The environmental monitoring provides relevant information about the temperature and luminosity level, which are fundamental to ensure the reliable control of lighting and HVAC systems. The luminosity level can be used to control part of the lighting, turning it on only when the luminosity is below a predefined level, avoiding a control just based on the time of day, which does not take into account the variation of sunshine hours between different months or even just the different luminosity levels in different hours and different days. The HVAC systems can be turned off in periods without users, using the people flow monitoring, but the most important control can be defined based on the temperature. HVAC systems can be turned off during short periods without a relevant impact on the thermal comfort, but such periods can be longer or shorter depending on the temperature values, namely, if there are extreme or mild values. Therefore, the temperature information of the HVAC system can be used to define the maximum period of turning off the HVAC systems. Such control ensures that the lighting and HVAC systems are used only when actually needed. This impact was evaluated by assessing previous load diagrams from a period prior to the implementation of the platform, and it was concluded that about 15% of demand (that occurred in hours without activity in the building) could be avoided with the implemented services.

The option of controlling the HVAC systems can be implemented to ensure objectives, such as the matching between generation and demand, reduction in costs, and provide demand response services. The matching between the generation and consumption at the building level is important to ensure the self-consumption of the PV generation, avoiding the constant power flow between the building and the grid with the associated losses and costs. Therefore, in periods of high demand, one option to improve the matching is by decreasing the demand by turning off part of the HVAC systems during a short period. On the contrary, in periods of high generation, the needed HVAC systems can be turned on earlier to ensure the pre-heating and pre-cooling of the building, using the available PV generation.

Such control also has an impact from the costs point-of-view, because a decrease in the power flow between the building and the grid due to a higher matching between the generation and demand leads to a lower cost, because the tariffs for the energy consumed from the grid are much higher than the tariffs for the energy injected into the grid. Additionally, the control can also be used only from the cost point-of-view, turning off some HVAC systems to decrease the demand during periods of higher tariffs. On the contrary, during periods of lower tariffs, the HVAC systems can be turned on to ensure the preheating or precooling at a lower cost. A typical application is the pre-heating in the early morning with off-peak tariffs, avoiding a higher demand due to the on-peak hours during the morning [47]. An additional option in a future scenario would be to use the platform to provide demand response services to the distribution system operator (DSO), increasing or decreasing the demand during short periods required by the DSO. Such services mainly rely on a future scenario after the installation of the designed PV system. The platform already ensures all the required data and control capabilities to implement such services, but such services can only be validated in a scenario with a large PV system able to create periods of generation surplus or of high variability that needs to be compensated with the demand.

## 7. Conclusions

This paper presents an integrative perspective to achieve a sustainable energy campus, with energy and IoT technologies. Firstly, a PV system with battery storage was designed to ensure a high share of demand with local generation, and then an IoT platform was proposed and deployed to monitor and control the demand.

The PV generation potential was assessed, with a designed PV system with 1.22 MWp able to ensure a generation level equivalent to 44% of the actual electricity demand. The design also included lithium-ion batteries with a total capacity of 480 kWh, with the objective to increase the self-consumption to 86.4%. Such an energy storage system can also be used to ensure the minimization of electricity costs, by storing energy in the periods with the lowest cost.

The designed IoT platform ensures not only the monitoring of demand and generation at the building level, but also for individual electrical systems, namely lighting and HVAC. Such a platform was implemented at a low cost and could easily be integrated into existing buildings without major installation requirements. From the scientific point-of-view, the main original contribution is the implementation of smart energy services with a low-cost platform. Such services ensure not only the monitoring of demand, contributing to identifying unnecessary consumption, but also the control, enabling the implementation of automatic controls to ensure the management of loads based on the time of day, type of day, season, presence of users, luminosity, and temperature. This is crucial not only to ensure energy savings, but also to control the demand to increase the matching between generation and demand, adapt the demand to the periods with lower tariffs, or to provide demand response services.

The designed generation system and IoT platform ensure the increase in energy sustainability in university campuses. Simultaneously, they ensure the smartness upgrade of existing buildings through innovations for legacy equipment in a cost-effective and reliable way. All in all, a platform such as this represents a remarkable test bench that would allow evaluating innovative technologies and services. The IoT platform was designed for university campuses, but could also be used in any large public or commercial building.

## Figures and Tables

**Table 1:** Comparison of main implementations in the literature.

Ref	Implementation	Limitations
[1]	Design of service discovery and perceptual data fusion algorithms for smart campuses	Implemented to provide services to the users and not for energy management
[9,10,11]	Design and implementation of photovoltaic (PV) technology and a battery storage system in a microgrid at building or campus level	There is no monitoring and control of demand to improve the self-consumption of PV generation
[13]	Design of an internet of things (IoT)-based model and implementation of environmental monitoring, car parking, and smart canteen	The monitoring and control of electricity demand, as well as the monitoring of PV generation, were not included
[14]	Design of a smart campus with geospatial information, control attendance, library, canteen and bus management	The monitoring and control of electricity demand, as well as the monitoring of PV generation, were not included
[15]	Implementation of an IoT platform for the flow of people and environmental monitoring	The monitoring and control of electricity demand, as well as the monitoring of PV generation, were not included
[16]	Implementation of the monitoring and control of the campus distribution network	The monitoring and control is at the distribution level (substations) and not for the individual consumption or generation
[17]	Implementation of physical demonstration sites for smart grids and smart buildings	Implementation at the distribution level and on a residential building in the campus, but not in the campus buildings
[18]	Implementation of the monitoring of renewable generation, global demand, and demand in buildings	The monitoring was implemented at the building and not at the system level and there is no demand control
[19]	Implementation of an energy management system that monitors the energy load and controls the lighting and heating, ventilation, and air conditioning (HVAC)	There is no monitoring of generation, being the control of demand only based on weather data
[21]	Implementation of an IoT and telecommunication architecture with monitoring and control of loads	There is no monitoring of generation, with the control of demand only based on weather data
[22]	A multidimensional situational information fusion mechanism is used to obtain a real-time situation and equipment control	There is no monitoring of generation, with the control of demand only based on weather data
[25]	Multisite IoT deployment aiming at enabling IoT-based energy awareness and sustainability lectures	Just provides electricity demand and weather data for awareness proposes, without any control option

**Table 2:** Quantity of PV panels and power.

Building	Number on Roof	Number on Facade	Total
A–D	2820	150	2970
B–C	1036	496	1532
Total	3856	646	4502

**Table 3:** Characteristics of the PV system.

Parameter	Value
Total Number of PV Modules	4502
Peak power	1.22 MWp
Number of PV inverters	72
Nominal AC power of the PV inverters	1.17 MW
AC active power	1.17 MW
Active power ratio	96.2%
Annual energy yield	1935.89 MWh
Energy usability factor	99.99%
Performance ratio	86.2%

**Table 4:** Distribution of PV energy and consumption.

Parameter	Value
Annual Energy Consumption	4398 MWh
Annual energy yield	1936 MWh
Grid feed-in	330 MWh
Purchased electricity	2792 MWh
Self-consumption	1606 MWh
Self-consumption quota (in % of PV energy)	83.0%
Self-generation (in % of energy consumption)	44.0%
Self-sufficiency quota (energy consumption in %)	36.5%

**Table 5:** Distribution of PV energy and consumption (scenario with energy storage)**.**

Parameter	Value
Annual Energy Consumption	4398 MWh
Annual energy yield	1936 MWh
Grid feed-in	263 MWh
Purchased electricity	2732 MWh
Self-consumption	1673 MWh
Self-consumption quota (in % of PV energy)	86.4%
Self-generation (in % of energy consumption)	44.0%
Self-sufficiency quota (energy consumption in %)	37.9%

**Table 6:** Technology comparison for short/medium coverage [33].

	Bluetooth	Wi-Fi	RFID IoT	ZIGBEE
**Work Frequency**	2.4 GHz	2.4 GHz	125–134 kHz	2.4 GHz 868/915 MHz
**Coverage**	10–30 m	30–100 m	10–20 cm	30–75 m
**Transmission Speed**	1 Mbps	100+ Mbps	200 bps/1 kbps	20/40/250 kbps
**Power Consumption**	Low	High	Low	Low
**Availability**	High	High	Low	Low
**Number of Elements**	8	50–200	1	255–65,000
**Cost**	Low	Low	High	High
